# Hemispheric Specialization for Processing the Communicative and Emotional Content of Vocal Communication in a Social Mammal, the Domestic Pig

**DOI:** 10.3389/fnbeh.2020.596758

**Published:** 2020-11-20

**Authors:** Lisette M. C. Leliveld, Sandra Düpjan, Armin Tuchscherer, Birger Puppe

**Affiliations:** ^1^Institute of Behavioural Physiology, Leibniz Institute for Farm Animal Biology (FBN), Dummerstorf, Germany; ^2^Institute of Genetics and Biometry, Leibniz Institute for Farm Animal Biology (FBN), Dummerstorf, Germany; ^3^Behavioural Sciences, Faculty of Agricultural and Environmental Sciences, University of Rostock, Rostock, Germany

**Keywords:** acoustic communication, conspecific calls, ear preference, hemispheric asymmetry, orienting bias, domestic pig, auditory lateralization

## Abstract

In humans, speech perception is lateralized, with the left hemisphere of the brain dominant in processing the communicative content and the right hemisphere dominant in processing the emotional content. However, still little is known about such a division of tasks in other species. We therefore investigated lateralized processing of communicative and emotionally relevant calls in a social mammal, the pig (*Sus scrofa*). Based on the contralateral connection between ears and hemispheres, we compared the behavioural and cardiac responses of 36 young male pigs during binaural and monaural (left or right) playback to the same sounds. The playback stimuli were calls of social isolation and physical restraint, whose communicative and emotional relevance, respectively, were validated prior to the test by acoustic analyses and during binaural playbacks. There were indications of lateralized processing mainly in the initial detection (left head-turn bias, indicating right hemispheric dominance) of the more emotionally relevant restraint calls. Conversely, there were indications of lateralized processing only in the appraisal (increased attention during playback to the right ear) of the more communicative relevant isolation calls. This implies differential involvement of the hemispheres in the auditory processing of vocalizations in pigs and thereby hints at similarities in the auditory processing of vocal communication in non-human animals and speech in humans. Therefore, these findings provide interesting new insight in the evolution of human language and auditory lateralization.

## Introduction

The study of vocal communication in non-human animals can provide useful information to improve our understanding of the evolution of human speech and language (Fitch, 2005; Beckers, 2011). Specifically, research on auditory perception of vocalizations in non-human animals can help in tracing the roots of the cortical processing of linguistic and paralinguistic content, such as the emotional state and identity of the sender, in human speech (Ghazanfar and Hauser, 1999; Belin, 2006). One of the specific features of human speech perception is that it is dominated by the left hemisphere (Tervaniemi and Hugdahl, 2003). This hemispheric specialization may have originated from left hemisphere dominance in the auditory perception of conspecific calls in non-human animals (reviewed by Ocklenburg et al., 2013). Although the dominance of the left hemisphere in conspecific communication is suggested to stem from specialization of the left hemisphere for the processing of rapid temporal sound changes (Zatorre and Gandour, 2008), some evidence suggests that the communicative relevance (or “meaning”) of the sound plays an important role (Petersen et al., 1984; Yasin, 2007). For instance, in some species, there are reports of left hemisphere dominance in perceiving heterospecific calls that nevertheless have communicative relevance, such as for dogs listening to human speech (Ratcliffe and Reby, 2014). Conversely, the communicative relevance of conspecific calls may be shaped by experience (Poremba et al., 2013) such that left hemisphere dominance for listening to certain calls may be restricted to some receivers, e.g., mother mice listening to pup calls (Ehret, 1987).

Apart from the processing of communicative information, other cognitive processes that are involved in the perception of vocal communication are also lateralized. For instance, in humans, the right hemisphere plays a dominant role in the perception of emotional prosody (Lindell, 2006). Additionally, the experience of emotions, which can be triggered by the perception of sounds, is asymmetrically processed. For instance, it is assumed that positive emotions are experienced with left hemisphere dominance and negative emotions with right hemisphere dominance in both humans and other vertebrate species (reviewed by Demaree et al., 2005; Leliveld et al., 2013). Indeed, some studies have found evidence of right hemisphere dominance in the perception of sounds that are associated with strong negative emotions, e.g., in dogs (Siniscalchi et al., 2008), frogs (Xue et al., 2015) and cats (Siniscalchi et al., 2016). Thus, lateralized auditory perception can reflect a combination of lateralized communicative and emotional processing. However, still much is unknown about the interaction between these lateralized cognitive processes during the auditory perception of vocal communication in non-human animals. For instance, most previous findings in non-human subjects have been based on head-turning biases (for reviews see Teufel et al., 2010; Ocklenburg et al., 2013), where it is assumed that turning one ear toward the source of a sound creates an auditory-input bias to the contralateral hemisphere. However, this behavioural expression of auditory lateralization is suggested to reflect only the initial detection of a biologically important sound that is directed by the inferior colliculus of the midbrain (Casseday and Covey, 1996; Teufel et al., 2010). It is therefore still unclear how auditory lateralization may be shaped by the different cognitive processes involved in the subsequent appraisal, which would involve also higher levels of cortical processing. In this study, we therefore aimed to investigate the roles of lateralized communicative and emotional processing in the initial detection and subsequent appraisal of vocal communication in a social mammal.

As highly vocal and social mammals (Kiley, 1972), pigs are an interesting model for studying auditory perception of vocalizations. To explore the effect of the communicative and emotional relevance of pig vocalizations on their lateralized processing, we focused on vocalizations from two different contexts: social isolation and physical restraint. Social isolation is an aversive situation for pigs, given their social nature, and vocalizations produced in this context are assumed to have a relatively strong communicative function, i.e., re-establishing contact with group members (Kiley, 1972; Marchant et al., 2001; Leliveld et al., 2017). Physical restraint resembles capture by a predator, and vocalizations produced in this context are assumed to have a relatively strong emotional function, i.e., sharing the emotional state of the sender (Weary and Fraser, 1995). Thus, based on the insight gained from previous studies (Kiley, 1972; Weary and Fraser, 1995; Marchant et al., 2001; Leliveld et al., 2017), it can be assumed that calls from these two contexts differ in both communicative and emotional relevance. To validate this assumption, we first confirmed the communicative and emotional relevance of calls from these contexts for our specific subject group, young male pigs, based on acoustic analyses and the behavioural and cardiac responses to binaural playback of these calls (part A). For studying auditory lateralization during the initial detection we used the already mentioned head-turn paradigm (Teufel et al., 2010; Ocklenburg et al., 2013). To gain insight into the hemispheric dominance in the subsequent behavioural and physiological appraisal of the sounds, we also presented the playbacks monaurally through earphones (Petersen et al., 1984) (part B). This reduces the sensory input to the ipsilateral hemisphere (Vallortigara, 2000). To account for possible baseline hemispheric dominance in auditory processing, we used a non-biological sound as a control. Since we hypothesize that the human auditory lateralized perception of the communicative and emotional content of speech originated from similarly lateralized auditory processing of vocalizations in non-human mammals, we expected that the communicative relevance of the social isolation calls would result in right-ear (indicating left-hemisphere) dominant auditory processing of these calls, while the (negative) emotional relevance of the restraint calls would result in left-ear (indicating right-hemisphere) dominant auditory processing of the calls.

## Methods

### Animals and Housing

The study was conducted at the Experimental Facility for Pigs at the Leibniz Institute for Farm Animal Biology in Dummerstorf, Germany. In total, 121 uncastrated male German Landrace pigs (aged 4–6 weeks) were used in this experiment: 49 for the recordings (3 replicates), 36 for binaural playback (3 replicates; part A) and 36 for monaural playback (3 replicates; part B). Upon weaning at 28 days of age, the subjects were selected based on their weight (>6 kg), health and heritage (avoiding full siblings where possible) and rearranged into an all-male group. The pigs that were used for the recordings were housed in a group of 10 to 12 pigs. The pigs that were used for the tests were housed in a group of 14 pigs (consisting of 12 subjects and 2 randomly selected reserves). The subjects were housed in a weaner pen (3 × 2 m with fully slatted plastic floors and a solid heated area in the middle). Water and food (Trede und von Pein GmbH, Itzehoe, Germany) were available *ad libitum*. Physical enrichment was provided in the form of a chew toy and rags. After testing was finished, the subjects stayed in the experimental facility.

### Experimental Setup

The recordings and playback experiments were all performed in the same experimental arena measuring 3 × 3 × 1.25 m, situated in a sound-attenuated experiment room. Above the centre of the arena were a camera (CCTV Color Camera YC 3189, B and S Technology GmbH, Eutin, Germany) connected to a security video network and a microphone (Sennheiser ME64/K6) connected to a recorder (Marantz PMD 670; sampling rate: 44.1 kHz, accuracy: 16 bit, mono). The arena was cleaned briefly between sessions and thoroughly at the end of each day.

### Recordings and Playback

The recordings were made in two consecutive contexts, social isolation and physical restraint. Each subject was fitted with a heart rate measurement belt (heart rate results not presented herein) and left in the arena for 5 min (social isolation). After this, the subject was fitted with a dog harness and fixed in a restraint stand (Düpjan et al., 2011) in the centre of the arena for 5 min (physical restraint). The sound recordings were saved in .wav format.

Thirty-six sections of 10 s each were selected from the isolation and restraint recordings (sound files S1 and S2). Suitable sections (entire calls and intervals <2 s) for both isolation and restraint were found for only 27 subjects. Therefore, a second pair of sections was included from each of 9 randomly selected subjects. Thirty-six control sections were generated in Adobe Audition 3.0 (Adobe, San Jose, USA). Each one was composed of a 500 Hz tone with a temporal pattern matching both the isolation and restraint sections of each caller. To compose the temporal patterns of the control sections, the call number and means and standard deviations of call durations and intervals of the isolation and restraint sections were entered in the DATA step of SAS to generate random duration and interval lengths. The 10 s sections were pseudo-randomly combined to make 36 1 min playback stimuli. The order of sections within the playback stimulus was balanced across the playback stimuli, and no caller was used twice within the same playback stimulus to avoid pseudo-replication. Additionally, playback stimuli were balanced as much as possible for the sum of call duration and call number (for isolation).

**Figure 1:**
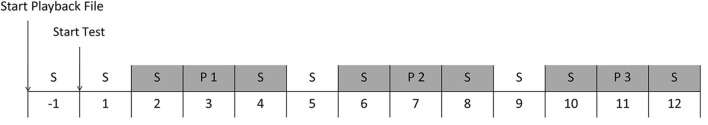
Schedule of one playback session. The numbers indicate the minutes from the start of the test. Grey areas indicate the minutes where behaviour and heart rate were analyzed. S = Silence, P1 = Playback stimulus 1, P2 = Playback stimulus 2, P3 = Playback stimulus 3.

In the playback experiments (part A and B), three playback stimuli (one of each type: isolation calls [IC], restraint calls [RC] and control sound [CS]) were played during a 12 min session (Figure 1). The playback stimuli were combined with silence intervals in a single playback file, with the playback stimuli starting at 2, 6, and 10 min from the beginning of the session. The order of the playback stimuli and playback modes (for part B; left ear, right ear or both ears) were balanced across individuals. All playback stimuli were produced in stereo, whereby in the case of monoaural presentation, only the left or right channel contained the sound. Since the restraint calls are naturally of a higher amplitude than isolation calls and since call amplitude also encodes information on the emotional state (Manteuffel et al., 2004; Briefer, 2012), we decided to adapt the volume of the playback as little as necessary. The volume of the playback was adapted only to the extent that the isolation calls were of the volume of a pig in the same room (mean volume: 68.7 dB), while the volume of the restraint calls had to be lowered to stay below the human pain level when presented through earphones (mean volume: 82.9 dB). The mean volume of the control sections was intermediate (75.4 dB) between the isolation and restraint calls.

### Handling and Habituation

Before the playback experiments started, the subjects were first handled for 3 days (days 5–7 after weaning), with two 1 h handling sessions per day. During these sessions, the subjects were habituated to the presence of the experimenter and were regularly touched on the belly and ears to reduce the stress during the fixing of the heart rate measurement belt and ear phones before the testing. To habituate the subjects to the control sound, we played the 36 control playback stimuli in the pen on days 5 to 7 after weaning and every day before the playback experiment (part A: day 14 after weaning; part B: days 14, 16 and 18 after weaning) from 7:30 to 13:15. Each day, the pigs heard 12 control playback stimuli at varying intervals (15–45 min). The stimuli were played at a mean volume of 82.2 dB from a laptop connected to the 3 front speakers of a surround sound speaker system (Logitech Z506), which were positioned in different corners of the pen. On days 12 and 13 after weaning, we habituated the pigs to the procedure of the playback experiment by performing the entire procedure except for the playback. On day 14, they had 1 day of rest before the playback experiment started.

### Playback Experiments

For the playback experiments, the pigs were fitted with a heart rate measurement belt (Polar WearLink with W.I.N.D. sensor, wirelessly connected to an RS800CX monitor; Polar Electro Oy, Kempele, Finland) and earphones (Panasonic RP-HS46E-W Slim Clip on Earphones, Panasonic, Kadoma, Japan) that were fixed to the ears with self-adhesive bandages (Fixomull stretch, BSN medical GmbH, Hamburg, Germany). The earphones were connected to an MP3 player (San Disk 8GB Sansa Clip plus; Western Digital, San Jose, USA) in a small backpack on their back. The playback file and heart rate measurements were started simultaneously, and the beep of the heart rate monitor was later used to synchronize the playback file and heart rate measurements with the video recordings. In part A, the pigs heard 3 playback stimuli (1 IC, 1 RC and 1 CS in a pseudo-randomized and balanced order) in both ears during a session of 12 min (on day 15 post weaning). In part B, the session was repeated 3 times (on days 15, 17 and 19) to present the subjects with 3 playback stimuli (1 IC, 1 RC and 1 CS) × 3 playback modes (left ear, right ear, both ears) in a pseudo-randomized and balanced order.

### Acoustic Analyses

All calls from the 72 selected 10-s sections (36 per recording context) that had no background noise were analyzed in Avisoft-SAS Lab Pro (Version 5.2.05; Avisoft Bioacoustics, Glienicke, Germany) according to the methods described in Leliveld et al. (2017). A short description of the settings used and parameters measured is provided in the Supplementary Material. In total, 222 calls in the IC sections and 68 calls in the RC sections could be analyzed. Twenty-one calls consisted of two distinctly different acoustic structures (mainly grunt-squeals), which were analyzed separately.

### Behavioural and Heart Rate Analyses

The behaviour and heart rate of the pigs in the minutes before, during and after the playback stimuli were analyzed (3 min in total). The following behaviours were recorded in Observer XT (version 12; Noldus Information Technology, Wageningen, The Netherlands): locomotion, standing/sitting, lying down, escape attempt, exploration, freezing and vocalization (continuous sampling; see Supplementary Table 1). In part A, the first head turn to the left or the right (> 45°) during playback was also recorded. Observations were made by two observers who were blind to the playback procedure (inter-observer reliability, based on 6 entire sessions: kappa based on all scored behaviours = 0.82).

Using Polar Precision Performance SW (version 4.03.040), R-R intervals (inter-beat intervals) were measured. The resulting data were corrected for artefacts (settings: very low sensitivity, peak detection on, minimal protection zone of 20) under visual inspection, excluding sections with > 10% artefacts, gaps of > 3 s and linear development for ≥ 5 consecutive R-R intervals (details described in Leliveld et al., 2016). The mean heart rate (HR) and the root mean square of successive differences between inter-beat intervals (RMSSD; indicating vagal activity) were calculated in 10 s intervals (Düpjan et al., 2011; Leliveld et al., 2016). Means of the resulting values were then calculated for each minute.

### Statistical Analyses

The data were analyzed using SAS (version 9.4; SAS Institute Inc., Cary, NC, USA). Call types were determined using a cluster analysis, according to the methods described in Leliveld et al. (2016). Nine parameters (duration, duration to maximum, peak frequency, the minimum and maximum frequency, Q25, Q75 [first and third quartile in the energy distribution across the frequency range], F2 [frequency of the 2nd peak] and SD PF [the standard deviation of the peak frequency]) were entered in the FASTCLUS procedure (MAXITER: 100, STRICT: 5). The proportional use of the call clusters (number of calls per cluster/total number of analyzed calls) was compared between the IC and RC sections with a generalized linear mixed model analysis (GLIMMIX procedure; distribution: Poisson; link function: log; repeated measurements were taken into account; block-diagonal residual covariance structure: compound symmetry) with replicate and context as fixed factors. The acoustic parameters were compared between the IC and RC sections with a generalized linear mixed model analysis (GLIMMIX procedure; distribution: normal; link function: identity; repeated measurements were taken into account; block-diagonal residual covariance structure: compound symmetry) with replicate, context and cluster as fixed factors and calls as a random factor. Multiple pairwise comparisons were made with the Tukey-Kramer test. Parameters that correlated strongly with other parameters (i.e., bandwidth, Q50 and entropy) were not included in this analysis.

A visual inspection of the distribution of latencies to turn the head (rounded up to the next second) across time showed that the number of head turns peaked during the 3rd second and then dropped during the 4th second. Therefore, only head turns within 3 s were included in the analysis for head-turning biases. The numbers of right and left head turns were then compared using a binomial test (FREQ procedure) for each stimulus type separately.

For the cardiac and behavioural measurements, we calculated differences to baseline (minute before playback) for the values of the minute during playback and the minute after playback. For part A, the effect of stimulus type on the cardiac and behavioural response was analyzed using a generalized linear mixed model analysis implemented by the GLIMMIX procedure (distribution: normal; link function: identity) with replicate, stimulus type and minute as fixed factors; playback order as a random factor; and subject as a repeated factor (block-diagonal residual covariance structure: unstructured). Cardiac data were analyzed with locomotion as a covariable. Multiple pairwise comparisons were made with the Tukey-Kramer test. The least-square means were tested against zero using an approximate *t*-test in GLIMMIX to determine whether the difference compared to the minute before playback is significant. For part B, the effects of playback mode, stimulus type and their interactions on cardiac and behavioural response were analyzed in the GLIMMIX procedure using a generalized linear mixed model analysis (distribution: normal; link function: identity) with replicate, stimulus type and ear as fixed factors; session and playback order as random factors; and subject as a repeated factor (block-diagonal residual covariance structure: compound symmetry). Multiple pairwise comparisons were made with the Tukey-Kramer test. The least-square means were tested against zero using an approximate *t*-test.

## Results

### Acoustic Properties of Playback Stimuli

Using the FASTCLUS procedure, we identified two call clusters (Cubic Cluster Criterion = 107.691, pseudo F statistic = 275.19). The first cluster counted 200 calls and had lower frequency values (low frequency call elements, mean peak frequency = 211.40 Hz) than the second cluster that counted 104 calls (high frequency call elements, mean peak frequency = 2742.31). The proportion of low frequency call elements was significantly higher in the IC sections than in the RC sections [*F*_(1,39.9)_ = 68.12, *p* < 0.001; Supplementary Table 2], while the proportion of high frequency call elements was higher in the RC sections [*F*_(1,26.8)_ = 108.73, *p* < 0.001]. Replicate had a significant effect on HNR [harmonic-to-noise-ratio; *F*_(2,14.8)_ = 6.27, *p* = 0.011]. Context had a significant effect on HNR [*F*_(1,246.8)_ = 4.30, *p* = 0.039], minimum frequency [*F*_(1,194.1)_ = 5.63, *p* = 0.019], maximum frequency [*F*_(1,244.0)_ = 4.74, *p* = 0.030] and Q25 [*F*_(1,247.1)_ = 6.69, *p* = 0.010] and there were significant cluster x context interaction effects on duration [*F*_(1,249.0)_ = 7.14, *p* = 0.008] and minimum frequency [*F*_(1,192.1)_ = 7.13, *p* = 0.008]. The high frequency call elements in the RC sections had a longer duration [*t*_(204.8)_ = −7.55, *p* < 0.001], lower HNR [*t*_(248.9)_ = 5.52, *p* < 0.001] and SD PF [*t*_(241.4)_ = 2.27, *p* = 0.024], as well as lower frequency values [peak frequency: *t*_(248.5)_ = −3.56, *p* < 0.001; minimum frequency: *t*_(192.2)_ = −9.06, *p* < 0.001; maximum frequency: *t*_(248.2)_ = −2.55, *p* = 0.011; Q25: *t*_(248.9)_ = −3.85, *p* < 0.001; Q75: *t*_(225.5)_ = −2.79, *p* = 0.006; F1 [frequency of 1st peak]: *t*_(248.6)_ = −4.43, *p* < 0.001] than in the IC sections.

### Part A (Binaural Playback): Biological Relevance

**Figure 2:**
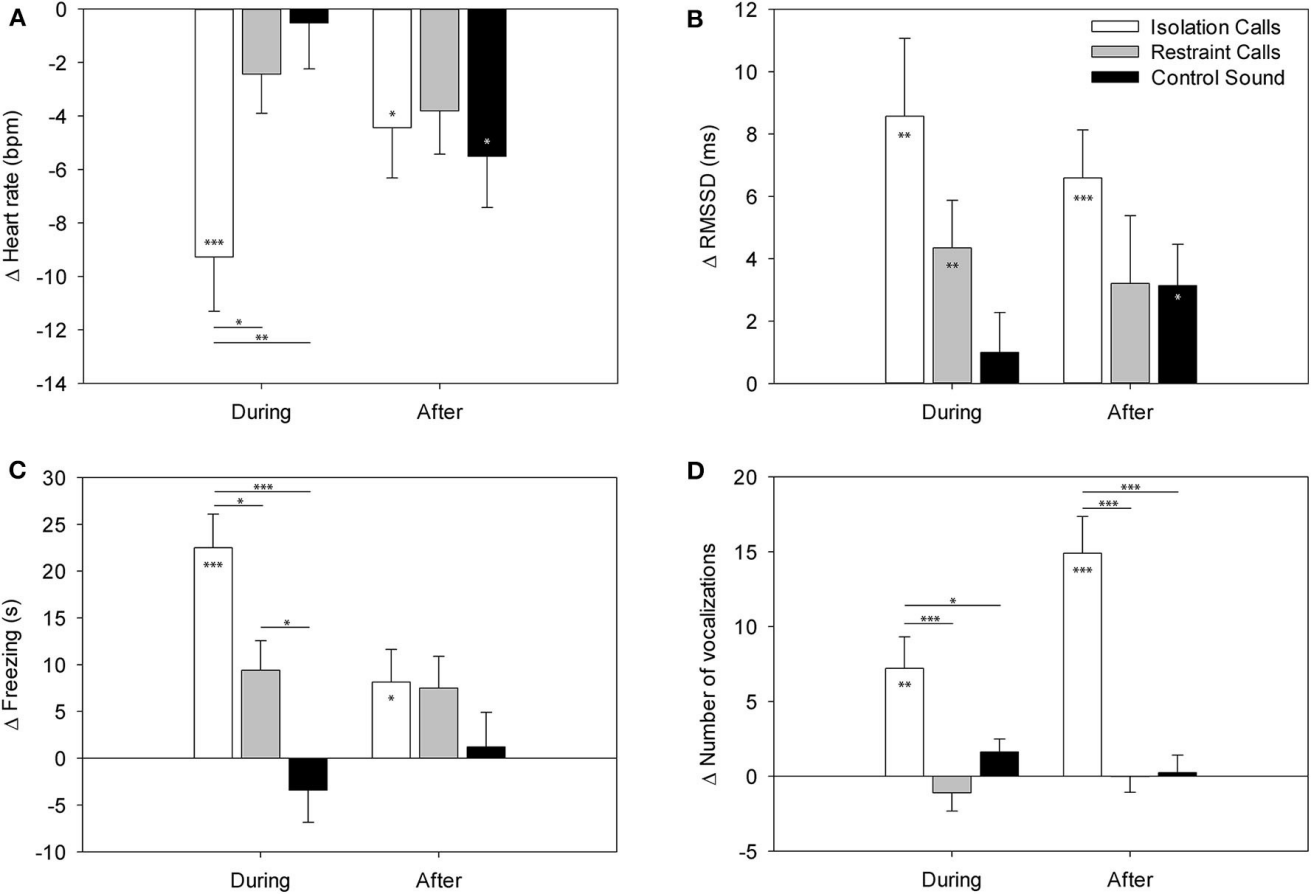
LS means ± standard error of **(A)** the heart rate, **(B)** the RMSSD, **(C)** freezing, and **(D)** number of vocalizations in the minutes during and after the playback in part A (binaural playback). Values are differences compared to the minute before playback. Asterisks within the bars indicate significant differences compared to baseline (minute before playback) and asterisks between bars indicate significant differences between the playback stimuli. ***p < 0.001, **p < 0.01, *p < 0.05.

The F-test results are shown in Table 1. The Tukey-Kramer test revealed that the HR during the IC playback was significantly more decreased than during the CS and RC playbacks [vs. CS: *t*_(9.2)_ = 3.25, *p* = 0.008; vs. RC: *t*_(5.3)_ = −2.95, *p* = 0.016; Figure 2]. Locomotion and exploration were also significantly more decreased during the IC playback than during the RC and CS playbacks [locomotion: vs. CS: *t*_(9.6)_ = 5.36, *p* < 0.001; vs. RC: *t*_(6.0)_ = −3.49, *p* = 0.004; exploration: vs. CS: *t*_(9.0)_ = 5.99, *p* < 0.001; vs. RC: *t*_(4.0)_ = −3.90, *p* = 0.001], while they were still more decreased during the RC playback than during the CS playback [locomotion: *t*_(6.2)_ = 2.59, *p* = 0.036; exploration: *t*_(7.7)_ = 2.99, *p* = 0.014]. Freezing, on the other hand, was significantly more increased during the IC playback than during the RC and CS playbacks [vs. CS: *t*_(9.2)_ = −5.15, *p* < 0.001; vs. RC: *t*_(6.2)_ = 2.92, *p* = 0.017], while it was more increased during the RC playback than during the CS playback [*t*_(6.0)_ = −2.86, *p* = 0.020]. Vocalizations were significantly more increased during and after the IC playback than during and after the RC and CS playbacks [vs. CS during: *t*_(35.3)_ = −2.47, *p* = 0.047; vs. RC during: *t*_(34.2)_ = 4.26, *p* < 0.001; vs. CS after: *t*_(34.2)_ = −6.34, *p* < 0.001; vs. RC after: *t*_(34.4)_ = 5.40, *p* < 0.001].

**Table 1:** F-values, DF (numerator degrees of freedom, denominator degrees of freedom), and *p*-values of the generalized linear mixed model analysis for part A (binaural playback).

	**Stimulus**			**Minute**			**Stimulus x Minute**			**Replicate**		
	**F**	**DF**	**p**	**F**	**DF**	**p**	**F**	**DF**	**p**	**F**	**DF**	**p**
Δ Heart rate [bpm]	1.84	2, 3.9	0.275	0.41	1, 29.2	0.526	**8.98**	**2, 29.8**	**<0.001**	1.75	2, 28.1	0.192
Δ RMSSD [ms]	2.56	2, 21.0	0.101	0.20	1, 25.0	0.660	1.35	2, 28.2	0.276	0.08	2, 18.4	0.922
Δ Locomotion [s]	5.79	2, 5.8	0.052	3.92	1, 34.9	0.056	**20.48**	**2, 35.0**	**<0.001**	**5.62**	**2, 30.7**	**0.008**
Δ Exploration [s]	**7.00**	**2, 4.2**	**0.046**	**16.87**	**1, 33.4**	**<0.001**	**16.06**	**2, 33.4**	**<0.001**	**2.68**	**2, 28.2**	**0.086**
Δ Freezing [s]	**5.38**	**2, 5.8**	**0.048**	**6.86**	**1, 32.8**	**0.013**	**16.22**	**2, 33.8**	**<0.001**	**5.01**	**2, 29.7**	**0.013**
Δ Vocalization [no.]	**17.73**	**2, 35.1**	**<0.001**	**6.33**	**1, 34.8**	**0.017**	**6.29**	**2, 35.4**	**0.005**	0.68	2, 33.1	0.513
Δ Escape attempts [s]	0.81	2, 201	0.445	0.04	1, 201	0.837	0.67	2, 201	0.512	0.05	2, 1	0.957

*Significant effects are highlighted in bold*.

The *t*-tests on the least square means revealed that the HR change showed significantly negative values during and after the IC playback [during: *t*_(11.4)_ = −4.56, *p* < 0.001; after: *t*_(7.9)_ = −2.36, *p* = 0.046] and after the CS playback [*t*_(8.5)_ = −2.89, *p* = 0.019], indicating a significant decrease compared to the minute before the playback (baseline). The RMSSD was significantly increased during the IC and RC playback [IC: *t*_(23.3)_ = 3.44, *p* = 0.002; RC: *t*_(32.3)_ = 2.85, *p* = 0.007] as well after the IC and CS playback [IC: *t*_(27.1)_ = 4.26, *p* < 0.001; CS: *t*_(21.2)_ = 2.36, *p* = 0.028]. Locomotion was significantly decreased during the IC and RC playback [IC: *t*_(10.2)_ = −6.66, *p* < 0.001; RC: *t*_(6.7)_ = −2.72, *p* = 0.031; Figure 2] and after the RC playback [*t*_(5.4)_ = −3.12, *p* = 0.024]. Exploration was significantly decreased during the IC and RC playback [IC: *t*_(10.4)_ = −7.77, *p* < 0.001; RC: *t*_(6.3)_ = −3.98, *p* = 0.007] and after the IC playback [*t*_(9.6)_ = −2.91, *p* = 0.016]. Freezing significantly increased during the IC and RC playback [IC: *t*_(10.1_ = 6.24, *p* < 0.001; RC: *t*_(6.1)_ = 2.96, *p* = 0.025] and after the IC playback [*t*_(9.6)_ = 2.32, *p* = 0.047]. Vocalizations were significantly increased during and after the IC playback [during: *t*_(35.0)_ = 3.45, *p* = 0.002; after *t*_(33.8)_ = 6.06, *p* < 0.001]. There were no significant differences in escape attempts and lying occurred too few times to be statistically analyzed.

### Part A (Binaural Playback): Auditory Lateralization

In response to the restraint calls, significantly more pigs turned their head toward the left than to the right (*p* = 0.027, *n* = 21; Table 2). No significant head-turn biases were found in response to the other types of playback stimuli.

**Table 2:** Number of head turns to the left or right within 3 s after the start of playback in part A (binaural playback).

**Stimulus type**	**No turn**	**Left**	**Right**	**Binomial test**
Isolation calls	22	8	6	*p* = 0.791, *n* = 14
Restraint calls	15	16	5	*p* = 0.027, *n* = 21
Control sound	14	9	13	*p* = 0.524, *n* = 22

*No turn indicates that no head turn was made within the 3 s after the start of playback. The binomial test outcomes are based on comparisons of left and right head turns*.

### Part B (Bi- and Monaural Playback)

There were no main effects of playback mode or stimulus type × playback mode on any of the measured parameters. However, Tukey-Kramer tests comparing playback modes within stimulus type revealed that freezing duration was significantly more increased during the IC playback to the right ear than to the left ear [*t*_(263.0)_ = −2.38, *p* = 0.048; Table 3]. The duration of escape attempts also decreased during IC playback to the right ear, which significantly differed from the IC playback to both ears [*t*_(263.6)_ = 2.99, *p* = 0.009].

**Table 3:** Least square means ± standard error of the heart rate and behavioural response during the 1-min playback of the **(A)** isolation calls, **(B)** restraint calls, and **(C)** control sound in part B (bi- and monaural playback).

**Playback mode**	**Both ears**	**Left ear**	**Right ear**
**A**	**ISOLATION CALLS**		
Δ Heart rate [bpm]	**–**3.12 ± 1.64	**−4.96 ± 1.69**	**−4.35 ± 1.66**
Δ RMSSD [ms]	**3.83 ± 1.45**	**3.91 ± 1.51**	**3.24 ± 1.48**
Δ Locomotion [s]	**–**3.76 ± 2.15	**−5.96 ± 2.12**	**−5.80 ± 2.13**
Δ Exploration [s]	–**10.02 ± 2.48**	**−6.29 ± 2.43**	**−8.69 ± 2.45**
Δ Freezing [s]	**9.05 ± 3.17^a^^b^**	**7.33 ± 3.12^a^**	**15.05 ± 3.14^b^**
Δ Vocalization [no.]	**6.19 ± 1.75**	**4.52 ± 1.71**	**4.54 ± 1.73**
Δ Lying down [s]	0.00 ± 1.08	**–**1.60 ± 1.06	0.30 ± 1.07
Δ Escape attempts [s]	0.11 ± 0.08^a^	0.00 ± 0.08**^a^^b^**	**−0.18 ± 0.08^b^**
**B**	**RESTRAINT CALLS**		
Δ Heart rate [bpm]	1.28 ± 1.61	**4.91 ± 1.63**	1.90 ± 1.54
Δ RMSSD [ms]	**–**2.02 ± 1.43	**–**0.73 ± 1.45	**–**1.72 ± 1.35
Δ Locomotion [s]	0.71 ± 2.15	3.20 ± 2.15	1.73 ± 2.11
Δ Exploration [s]	**–**0.55 ± 2.48	**–**0.68 ± 2.48	0.61 ± 2.41
Δ Freezing [s]	**–**4.37 ± 3.17	**−8.09 ± 3.17**	**–**4.50 ± 3.09
Δ Vocalization [no.]	1.63 ± 1.5	**6.00 ± 1.75**	2.39 ± 1.69
Δ Lying down [s]	1.29 ± 1.08	**–**0.55 ± 1.08	**–**0.57 ± 1.05
Δ Escape attempts [s]	0.00 ± 0.08	**–**0.07 ± 0.08	0.09 ± 0.07
**C**	**CONTROL SOUND**		
Δ Heart rate [bpm]	**5.02 ± 1.59**	**3.74 ± 1.61**	2.22 ± 1.66
Δ RMSSD [ms]	–**3.50 ± 1.41**	**–**1.08 ± 1.43	**–**2.06 ± 1.48
Δ Locomotion [s]	2.35 ± 2.11	4.26 ± 2.13	3.03 ± 2.16
Δ Exploration [s]	**6.83 ± 2.41**	3.20 ± 2.45	**7.86 ± 2.50**
Δ Freezing [s]	**−9.78 ± 3.09**	**–**7.06 ± 3.14	**−7.25 ± 3.19**
Δ Vocalization [no.]	**3.78 ± 1.69**	3.04 ± 1.73	2.01 ± 1.77
Δ Lying down [s]	0.69 ± 1.04	1.36 ± 1.07	**–**1.52 ± 1.10
Δ Escape attempts [s]	0.06 ± 0.07	0.00 ± 0.08	**–**0.01 ± 0.08

*Values are differences compared to the minute before playback. Bold values represent significant differences (t-test) compared to baseline (the minute before playback)*;

a, b *means with different superscripts in the same row differ significantly (Tukey-Kramer test)*.

Results of the *t*-tests on the least square means (compared to baseline) are mentioned only when they differed between the treatments (see Table 3 for full details). During the IC playback, the HR and locomotion significantly decreased when played to the right ear or to the left ear [HR: right ear: *t*_(13.8)_ = −2.63, *p* = 0.020; left ear: *t*_(15.0)_ = −2.93, *p* = 0.010; locomotion: right ear: *t*_(6.0)_ = −2.71, *p* = 0.035; left ear: *t*_(5.8)_ = −2.81, *p* = 0.032], and escape attempts significantly decreased during playback to the right ear [*t*_(67.2)_ = −2.40, *p* = 0.019]. During the RC playback to the left ear, HR and vocalizations significantly increased [HR: *t*_(13.1)_ = 3.01, *p* = 0.010; vocalizations: *t*_(19.9)_ = 3.43, *p* = 0.003], while freezing significantly decreased [*t*_(8.7)_ = −2.55, *p* = 0.032]. During the CS playback to both ears or to the left ear, HR significantly increased [both ears: *t*_(11.8)_ = 3.16, *p* = 0.008; left ear: *t*_(12.5)_ = 2.31, *p* = 0.038]. During CS playback to both ears, RMSSD significantly decreased [*t*_(12.8)_ = −2.48, *p* = 0.028], and vocalizations significantly increased [*t*_(17.5)_ = 2.23, *p* = 0.039]. During CS playbacks to both ears and to the right ear, exploration significantly increased [both ears: *t*_(10.2)_ = 2.84, *p* = 0.017; right ear: *t*_(11.8)_ = 3.14, *p* = 0.009], while freezing significantly decreased [both ears: *t*_(7.9)_ = −3.16, *p* = 0.014; right ear: *t*_(9.0)_ = −2.27, *p* = 0.050].

## Discussion

Using the example of young pigs, the approach of the study was to examine, whether the lateralized perception of human speech has its evolutionary roots in non-human mammals. In this case, the auditory processing should also be lateralized. During binaural and monaural presentation of conspecific calls recorded during isolation (mainly communicative calls) and physical restraint (mainly emotional calls) we found differential response patterns between call contexts and indications of ear advantages in the perception of these sounds. This implies hemispheric specialization in the auditory perception of pig vocalizations and suggests similar patterns of lateralized emotional and communicative processing between humans and non-human mammals.

The acoustic analyses of the playback stimuli, together with the behavioural and physiological response during binaural presentation, confirms our assumption based on previous studies that IC playbacks are of mostly communicative relevance, while RC playbacks are of more emotional relevance. The IC playbacks mainly consisted of low-frequency calls, which are likely used for trying to re-establish contact with group members (Kiley, 1972; Marchant et al., 2001; Leliveld et al., 2017), and they resulted in a greater attentive (e.g., lower HR and more freezing) and vocal response than CS playbacks. The RC playbacks mainly consisted of high-frequency calls, which are indicative of high (negative) arousal (Marchant et al., 2001; Leliveld et al., 2017) and therefore are likely used for sharing the emotional state of the sender (Weary and Fraser, 1995). Playback of RC resulted in an intermediate attentive response between IC and CS playbacks, which reflects the lesser ability/need for young piglets to act in a distress context (Düpjan et al., 2011) compared to an isolation context.

Binaural IC playbacks did not lead to a significant head-turn bias, indicating no clear hemispheric asymmetries. This is in contrast to most previous reports, where communication calls were usually, but not always, found to be processed with left hemisphere dominance (Ocklenburg et al., 2013). The head-turn bias, however, may merely indicate which hemisphere dominates in the initial detection of the call (Casseday and Covey, 1996; Teufel et al., 2010). Monaural playback, on the other hand, can provide insight into the roles of each hemisphere in the subsequent appraisal of a sound. Monaural IC playback to the right ear strengthened the increase in freezing duration compared to playback to the left ear (though not compared to both ears), which suggests that this action is guided by the left hemisphere. Freezing is often interpreted as an indicator of fear (Fureix and Meagher, 2015). However, since pigs were also found to freeze in response to play barks (Chan et al., 2011), this reaction may also merely reflect an attentional response. The decrease in escape attempts during playback to the right ear, which was significantly lower than for both ears, could be interpreted as decreased fear (Murphy et al., 2014). This could be the result of reducing the input to the right hemisphere (the left ear received no playback) because the right hemisphere is dominant in processing negative emotions (Leliveld et al., 2013). Although the other parameters did not show any lateralized effects, these results seem to hint that the left hemisphere guides attention to calls of communicative relevance, while the right hemisphere guides the fear responses to these calls.

Binaural RC playback led to a significant left head-turn bias, suggesting right hemisphere dominance in the initial detection of these calls. Since the restraint calls were found to be of more emotional than communicative relevance to young male pigs, these findings suggest that it may not be the communicative relevance (Petersen et al., 1984; Böye et al., 2005), but rather the emotional relevance (Scheumann and Zimmermann, 2008; Xue et al., 2015) that determines the lateralized perception of these calls. Monaural playback, however, resulted in no significant differences between playback modes. There were only subtle, indirect indications of hemispheric dominance in that monaural playback to the left ear resulted in some significant changes that were not found for playback to both ears or to the right ear. Playback to the left ear resulted in a significant increase in HR and vocalizations, combined with a decrease in freezing, which suggests a more aroused and active response (Manteuffel et al., 2004; von Borell et al., 2007). This response was absent during playback to both ears, which may be due to an inhibition of emotional responses by the left hemisphere (Andrew and Rogers, 2002). However, it is important to note that this is only indirect evidence, since differences between playback modes were not significant. Therefore, these findings need to be treated with caution. The CS playback did not evoke a significant head-turn bias, and although the monaural presentation revealed effects of playback mode, this could not be easily interpreted. Since the response of the subjects to the binaural playback of CS in part A indicated that this sound had no apparent communicative or emotional relevance to the subjects, our findings suggest that auditory processing is not a lateralized process in itself, but rather depends on the type of sound.

Together, these results seem to be in line with findings in humans (Buchanan et al., 2000; Lindell, 2006) and marmosets (Hook-Costigan and Rogers, 1998), suggesting that the left hemisphere has a processing advantage for the communicative relevance and the right hemisphere has a processing advantage for the emotional relevance of vocal communication in pigs. However, while we found evidence of lateralized processing of emotionally relevant calls mainly in the initial detection (head-turn bias) and only very subtle effects in the subsequent appraisal (monaural playback response), evidence regarding communicatively relevant calls was detected only in the subsequent appraisal. This difference between the initial detection and subsequent appraisal, may reflect that different levels of cortical auditory processing were involved. As mentioned before, the head turning toward a sound may be mediated by the inferior colliculus in the midbrain (Casseday and Covey, 1996; Teufel et al., 2010). The role of the inferior colliculus in auditory processing is suggested to mainly consist of a quick recognition of the acoustic features of sounds requiring immediate action (e.g., sounds indicating the presence of a predator) and then to direct a fixed action pattern (e.g., head turn) to quickly respond to this urgent situation (Casseday and Covey, 1996). Thus, at this level auditory processing seems to focus more on emotional relevance, while a more detailed extraction of communicative information requires the involvement of higher level, cortical structures (Kanwal and Rauschecker, 2014; Bodin and Belin, 2019), which become involved at a later stage, such as during the subsequent appraisal. This interpretation should be handled with caution though, since the subjects did not respond to the restraint calls with any other clear behavioural responses to a potential threat, although, as mentioned before, young piglets have a lesser need/ability to respond in a situation of distress (Düpjan et al., 2011). In the future, more detailed information on hemispheric asymmetries in the processing of conspecific calls may be provided using direct measures of brain activity (e.g., based on advanced imaging techniques like fMRI). However, since such techniques are still not easily available for pigs, the behavioural measures used in this study provide the most reliable indirect indicators of lateralized auditory processing.

Since for some species, an absence of head-turn biases in response to conspecific calls has been reported for juveniles (Hauser and Andersson, 1994; Böye et al., 2005), the lack of communicative lateralization in the initial detection of conspecific calls in our juvenile subjects may also be due to their immaturity. Some researchers suggested that this may reflect that juveniles have not yet fully learned the communicative significance of conspecific calls (Hauser and Andersson, 1994; Böye et al., 2005). However, since we found that isolation calls are of communicative significance to young male pigs, it seems rather that it is the lateralized initial detection of these calls that is not yet developed. In humans although both emotional and linguistic processing of vocal communication are rudimentary lateralized in new-borns (e.g., Dubois et al., 2009; Zhang et al., 2017) the development of lateralized processing can take several years (Rogers, 2014) and to our knowledge it is still unclear which process becomes lateralized earlier. However, it is suggested that at an early age vocal communication is mainly emotion-based (Kubicek and Emde, 2012) and therefore there may be a greater need for efficient (and therefore lateralized) processing of the emotional content. Indeed, the production of strong negative emotional calls, such as distress calls, was found to be lateralized in non-human primate infants (Wallez and Vauclair, 2012). However, to confirm whether the developmental stage of our subjects affected the results in this study, further research on pigs of different age classes is required. So taking the above mentioned limitations into account, our study provides new insight in the roles of lateralized emotional and communicative processing in the auditory perception of non-human vocal communication and hints at a predominantly emotional lateralization in the initial (possibly midbrain-driven) detection and a predominantly communicative lateralization in the (possibly cortex-driven) subsequent appraisal.

In summary, we found subtle indications of left hemisphere specialization for the processing of more communicative relevant calls and right hemisphere specialization for the processing of more emotionally relevant calls. However, we found evidence of lateralized processing of the emotional calls mainly in the initial detection, while evidence of lateralized processing of the communicative calls was detected only in the appraisal. This hints at an earlier (and therefore possibly brainstem level) auditory lateralization in processing the emotional content and a later (and therefore possibly cortical level) auditory lateralization in processing the communicative content of vocal communication in young pigs. Future studies focusing on cerebral activation in response to these vocalizations are needed to confirm this.

## Data Availability Statement

The raw data supporting the conclusions of this article will be made available by the authors, without undue reservation.

## Ethics Statement

The animal study was reviewed and approved by the ethics committee of the Federal State of Mecklenburg-Western Pomerania (LLAF M-V/TSD/7221.3-2-046/14).

## Author Contributions

LL, SD, and BP conceived and designed the experiments. LL and SD performed the experiments. LL and AT analyzed the data. All authors were involved in the preparation of the article.

## Conflict of Interest

The authors declare that the research was conducted in the absence of any commercial or financial relationships that could be construed as a potential conflict of interest.
