# Supplementary material for: Hemispheric Specialization for Processing the Communicative and Emotional Content of Vocal Communication in a Social Mammal, the Domestic Pig
Source: Front Behav Neurosci. 2020 Nov 20;14:596758. doi: 10.3389/fnbeh.2020.596758 (PMC7714956; doi:10.3389/fnbeh.2020.596758)
Supplement: Supplementary file 1 [file Data_Sheet_1.docx]

Supplementary Material

# Supplementary methods

## Acoustic analyses (additional information)

The spectrogram parameters were set at 1024 FFT (fast Fourier transform) length, Hamming window, and 50% window overlap, resulting in a frequency resolution of 43 Hz and a temporal resolution of 11.6 ms, and a high-pass cut-off frequency at 100 Hz (Leliveld et al., 2017). Calls were analyzed in the spectrogram window, using the ‘insert section label from marker’ function to select calls and the ‘automatic parameters measurement’ option for parameter extraction. The following parameters were measured: duration, duration from start to maximum amplitude (DurMax), peak frequency, the minimum and maximum frequency (threshold: -20 dB relative to peak frequency, “total” option activated) and resulting bandwidth, three quartiles that describe the distribution of energy over the frequency range (Q25 refers to the 1^st^; Q50 to the 2^nd^; and Q75 to the 3^rd^ quartile in the energy distribution across the frequency range), the number of peaks (above -20 dB, hysteresis: 10 dB), the frequency of the first two peaks (F1 and F2), the entropy, the harmonic-to-noise ratio (HNR) and standard deviation of the peak frequency (SD PF) across the call (Leliveld et al., 2017). Frequency parameters were log-transformed.

# References

Leliveld, L.M.C., Düpjan, S., Tuchscherer, A., Puppe, B. (2017). Vocal correlates of emotional reactivity within and across contexts in domestic pigs (*Sus scrofa*). *Physiol. Behav*. 181, 117–126. doi: 10.1016/j.physbeh.2017.09.010

# Supplementary Tables

**Table S1.** Ethogram of the analyzed behaviours. Locomotion, standing/ sitting, lying, escape attempts, vocalizations and first head turn were mutually exclusive behaviours. Exploration and freezing were analyzed separately.

| Behaviour | Type | Measurement | Definition |
| --- | --- | --- | --- |
| Locomotion | State event | Duration | Movement with at least two legs. |
| Standing/ sitting | State event | Duration | No locomotion. A minimum of 3 feet are touching the floor. All four legs, or at least the front two legs, stretched out and no contact between torso and floor. |
| Lying | State event | Duration | The subject lies with the whole belly or whole lateral part of the subject touching the floor. |
| Escape attempt | State event | Duration | Jumping at the wall or raising the front feet to the wall. |
| Exploration | State event | Duration | Manipulating the floor/ wall using the nose. |
| Freezing | State event | Duration | No movement by any body part for 1 second or longer. |
| Vocalization | Point event | Frequency | The subject vocalizes. Each single call is counted. |
| First head turn | Point event | Direction | The head position at the start of the playback is taken as a reference point. After starting the playback, the first head turn of more than 45 degrees to the left or right from the reference point is scored. |

**Table S2**. Least square means ± standard errors of call proportions and acoustic properties of the two call clusters per recording situations. DurMax refers to the duration from start to maximum amplitude, Q25 and Q75 refer to the 1st and 3^rd^ quartile in the energy distribution across the frequency range, F1 and F2 refer to the frequency of the first two peaks, HNR refers to the harmonic-to-noise ratio and SD PF refers to the standard deviation of the peak frequency across the call ^a, b^ means within a row and call type with different superscripts differ significantly (Tukey Kramer test).

| Call type | Low frequency | |  | High frequency | |
| --- | --- | --- | --- | --- | --- |
| Context | Isolation | Restraint |  | Isolation | Restraint |
| Proportion | 60.88 ± 0.07^a^ | 90.05 ± 0.02^b^ |  | 60.11 ± 0.02^a^ | 00.96 ± 0.08^b^ |
| DurMax [%] | 63.99 ± 1.89 | 94.30 ± 24.68 |  | 66.60 ± 4.15 | 64.50 ± 5.93 |
| Duration [s] | 60.21 ± 0.01 | 90.11 ± 0.18 |  | 00.08 ± 0.03^a^ | 00.45 ± 0.04^b^ |
| Peak frequency [Hz] | 62.26 ± 0.03 | 92.19 ± 0.31 |  | 03.07 ± 0.06^a^ | 03.38 ± 0.08^b^ |
| Minimum frequency [Hz] | 61.92 ± 0.03 | 91.91 ± 0.15 |  | 01.98 ± 0.02^a^ | 02.35 ± 0.04^b^ |
| Maximum frequency [Hz] | 62.89 ± 0.02 | 93.21 ± 0.21 |  | 03.91 ± 0.04^a^ | 04.06 ± 0.05^b^ |
| Q25 [Hz] | 62.39 ± 0.02 | 92.68 ± 0.18 |  | 03.20 ± 0.03^a^ | 03.41 ± 0.05^b^ |
| Q75 [Hz] | 63.04 ± 0.05 | 93.22 ± 0.25 |  | 03.77 ± 0.06^a^ | 03.97 ± 0.07^b^ |
| Number of peaks | 61.50 ± 0.11 | 93.21 ± 1.45 |  | 06.59 ±0.24 | 06.22 ± 0.35 |
| F1 [Hz] | 62.18 ± 0.02 | 92.11 ± 0.24 |  | 02.30 ± 0.04^a^ | 02.61 ± 0.06^b^ |
| F2 [Hz] | 32.72 ± 0.03 | 92.76 ± 0.27 |  | 02.98 ± 0.05 | 03.10 ± 0.07 |
| HNR | 33.50 ± 0.51 | 30.59 ± 4.99 |  | 33.60 ± 0.92^a^ | 25.58 ± 1.26^b^ |
| SD PF | 30.43 ± 0.02 | 90.55 ± 0.24 |  | 00.73 ± 0.04^a^ | 00.57 ± 0.06^b^ |
